# Chitosomes-In-Chitosan Hydrogel for Acute Skin Injuries: Prevention and Infection Control

**DOI:** 10.3390/md19050269

**Published:** 2021-05-12

**Authors:** Lisa Myrseth Hemmingsen, Kjersti Julin, Luqman Ahsan, Purusotam Basnet, Mona Johannessen, Nataša Škalko-Basnet

**Affiliations:** 1 Drug Transport and Delivery Research Group, Department of Pharmacy, University of Tromsø The Arctic University of Norway, Universitetsvegen 57, 9037 Tromsø, Norway; lisa.m.hemmingsen@uit.no (L.M.H.); luqmaan.ahsan@gmail.com (L.A.); 2 Research Group for Host-Microbe Interaction, Department of Medical Biology, University of Tromsø The Arctic University of Norway, Sykehusvegen 44, 9037 Tromsø, Norway; kjersti.julin@uit.no (K.J.); mona.johannessen@uit.no (M.J.); 3 IVF Clinic, Department of Obstetrics and Gynecology, University Hospital of North Norway, Sykehusvegen 38, 9019 Tromsø, Norway; purusotam.basnet@uit.no; 4 Women’s Health and Perinatology Research Group, Department of Clinical Medicine, University of Tromsø The Arctic University of Norway, Universitetsvegen 57, 9037 Tromsø, Norway

**Keywords:** chitosan-infused liposomes, chitosan hydrogel, membrane-active antimicrobials, bacterial eradication, acute wound management, *Staphylococcaceae*

## Abstract

Burns and other skin injuries are growing concerns as well as challenges in an era of antimicrobial resistance. Novel treatment options to improve the prevention and eradication of infectious skin biofilm-producing pathogens, while enhancing wound healing, are urgently needed for the timely treatment of infection-prone injuries. Treatment of acute skin injuries requires tailoring of formulation to assure both proper skin retention and the appropriate release of incorporated antimicrobials. The challenge remains to formulate antimicrobials with low water solubility, which often requires carriers as the primary vehicle, followed by a secondary skin-friendly vehicle. We focused on widely used chlorhexidine formulated in the chitosan-infused nanocarriers, chitosomes, incorporated into chitosan hydrogel for improved treatment of skin injuries. To prove our hypothesis, lipid nanocarriers and chitosan-comprising nanocarriers (≈250 nm) with membrane-active antimicrobial chlorhexidine were optimized and incorporated into chitosan hydrogel. The biological and antibacterial effects of both vesicles and a vesicles-in-hydrogel system were evaluated. The chitosomes-in-chitosan hydrogel formulation demonstrated promising physical properties and were proven safe. Additionally, the chitosan-based systems, both chitosomes and chitosan hydrogel, showed an improved antimicrobial effect against *S. aureus* and *S. epidermidis* compared to the formulations without chitosan. The novel formulation could serve as a foundation for infection prevention and bacterial eradication in acute wounds.

## 1. Introduction

Acute skin injuries, such as burns, cuts, or other trauma, are painful breaches of the skin. With the growing numbers of resistant pathogens, we need to prevent bacterial infections and treat these breaches timely and efficiently. Larger skin injuries such as burn trauma cause destruction of the first line of defence, impairing both the physical barrier and the immune system [1]. These entry points are leaving the patients more vulnerable to bacterial colonisation and infections [1]. Additionally, it is estimated that as much as 75% of attributable mortality in this patient group is linked to infections, making this the primary cause of death [2]. Here, skin and soft tissue infections (SSTIs) are the second leading healthcare-associated class following burn injuries [3] and one of the most common bacterial infections in the human population [4]. The burns are often prone to biofilm formation, increasing the complexity of the wounds and leading to chronicity [5]. The escalating threat of antimicrobial resistance and biofilm-producing strains influence the treatment outcome [6]. The incidents of burn injuries are ostensibly decreasing [7]; however, nearly 9 million injuries globally were related to fire, heat, or hot substances, according to the Global Burden of Disease 2017 study [8].

In pursuance of novel treatment options for burns and other acute wounds, formulations aiding both microbial eradication and the wound-healing process are highly desirable. Pharmaceutical technology and nanotechnology could be utilized to increase both these processes [9]. Herein, the selection of the materials exhibiting intrinsic wound healing, as well as antimicrobial properties, is fundamental. Chitosan, a natural, cationic polymer, derived from the deacetylation of chitin [10], has attracted attention as a biomaterial for wound management [11]. This bioactive polymer, found in marine crustaceans, fungi, and insects, is regarded both biocompatible and biodegradable [12,13] with confirmed intrinsic antimicrobial [14] and wound-healing properties [15]. As a result, chitosan has been utilized in the preparation of various pharmaceutical formulations, ranging from solid and semi-solid to liquid forms [16]. However, in topical skin therapy, lipid-based delivery systems, such as liposomes, are often particularly interesting because of their potential interaction with the skin structure [17] as well as being a solubilizer for substances with lowered solubility [18]. Moreover, the antimicrobial potential of the lipid-based vesicles, liposomes, can be enhanced by coating of their surface or inclusion of the bioactive polymers to both improve wound healing and antimicrobial properties [19]. The possibility to infuse liposomes with chitosan forming chitosomes was previously proposed by our group [20]. These novel vesicles were challenged against vaginal *Candida* infections and both chitosomes alone and chitosomes with incorporated metronidazole eradicated *Candida* [20]. These chitosomes, unlike many other nanoparticle-based formulations, were prepared through a rapid one-step method.

Considering the improved antibacterial action, combining chitosomes with membrane targeting antimicrobials could further increase the antimicrobial capacity through synergic effects on the bacterial membrane [21]. Chlorhexidine (CHX), a membrane active antimicrobial (MAA), is frequently used in the prevention of SSTIs and commonly used in burn units [22]. The main antibacterial mechanism of CHX is proposed to be destruction of the bacterial membrane; however, precipitation of the cytoplasm has been observed when CHX is administered in higher concentrations [23]. Furthermore, topical formulations of CHX are commonly used in combinational therapy for chronic wounds [24]. Exploiting the activity of MAAs, such as CHX, in combination with chitosan of higher molecular weight, affecting the bacterial membrane [25], could prove beneficial in bacterial prevention and eradication.

**Figure 1:**
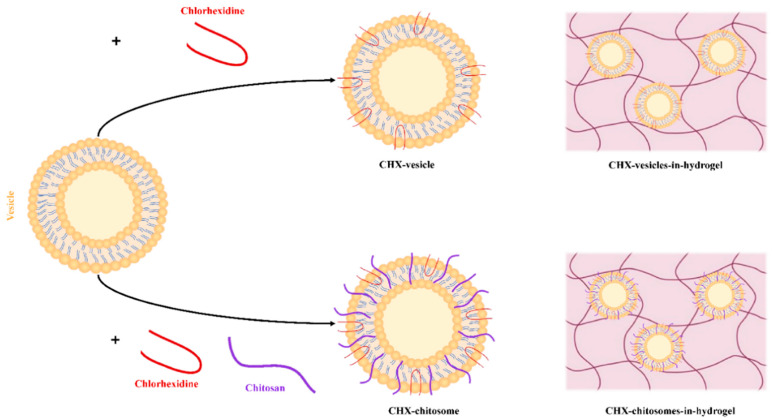
Illustration of the two types of vesicles utilized in the current study. In the top-half of the illustration, the CHX-vesicles (chitosan-free) both as vesicle alone and incorporated in hydrogel network are presented. In the bottom-half of the illustration, the chitosan-infused vesicles, chitosomes, with entrapped CHX are presented both as vesicles alone and incorporated in hydrogel.

Liposomal suspensions are not suitable for direct application onto the skin due to low viscosity and retention; this limitation is often solved by incorporating the vesicles into hydrogels [26]. In addition to serving as a vehicle for liposomes, the hydrogel could also provide an improved release profile and further increase accumulation of the antimicrobial compound in the wound area [27]. In this study, chitosan was selected as a hydrogel base due to its bioadhesive and biocompatible properties, which are suitable for pharmaceutical applications [28,29]. Moreover, we aimed to tailor the release of CHX to assure rapid and efficient microbial prevention and eradication. Although the hydrogel would swell to a certain degree in physiological fluids [30], to assure the fast release as well as prolonged retention on the skin, we combined chitosomes with chitosan hydrogels (Figure 1).

In our previous study, we utilized conventional liposomes as primary vesicles for CHX further incorporated in chitosan hydrogel for the treatment of chronic wounds. The novel formulation assured sustained CHX release [18]. However, that formulation would not be optimal for acute wound treatment. To modify the rate of the CHX release to achieve faster and efficient antimicrobial action, we propose chitosomes as primary vesicles for CHX. Andersen et al. showed an initial burst-release from their chitosomes and postulated that this effect might be due to the arrangement of the pharmaceutical compound in the bilayer [31]. In chitosomes, CHX is most likely incorporated within the bilayer and associated with the surface of chitosomes, allowing a faster initial release of CHX. Additionally, chitosan infused in the vesicles (chitosomes) is surface-available and has the possibility of closely interacting with the bacterial membrane immediately (Figure 1). These two factors could act in synergy, providing a faster onset of the antimicrobial action. Since most of the CHX is preserved within the bilayer of chitosomes, it could contribute to the long-term effect, similar to what has been previously confirmed for conventional CHX liposomes [18]. We hypothesized that combining CHX with chitosan-infused vesicles, chitosomes, could improve microbial eradication, and in a combination with the hydrogel network, serve as a promising platform for the prevention of bacterial colonization of acute wounds.

## 2. Results and Discussions

### 2.1. Vesicle Characteristics

Chitosan-based formulations could potentially support the wound-healing process in all stages of the complex healing cascade [32]. Additionally, hydrogels comprising this bioactive polymer could counteract the factors impairing healing processes by anti-inflammatory and antimicrobial actions [33]. Among all biomaterials, chitosan is one of the most frequently used ingredients in hydrogel preparation [34,35]; however, other formulations are also reported such as nanofibers [36] and nanoparticles [37]. Moreover, chitosan is often used as a coating material for vesicles [14]. In this study, we intended to exploit chitosan’s beneficial intrinsic properties in both the primary and secondary vehicle to maximize the potential treatment outcome. As this formulation is intended for topical therapy of skin burns and other acute wounds, lipid-based vesicles were selected as the primary vesicle.

#### 2.1.1. Vesicle Characteristics

The size and zeta potential of vesicles are known to influence the characteristics of the hydrogel [38] and the treatment outcome. Consequently, we evaluated the size, zeta potential, CHX entrapment, and pH of the vesicles (Table 1). These properties are influenced by the method of preparation. The one-pot method generates larger vesicles with broader size distribution [31]; therefore, probe sonication was utilized to reduce the vesicle size. The vesicle size was additionally influenced by the incorporation of CHX. A single sonication cycle was sufficient to reach the intended size. For comparison, to reach the same vesicle size, the empty vesicles required several sonication cycles. Our targeted vesicle size was around 200 nm, which was the lower end of the optimal vesicle size range intended for dermal delivery [39].

**Figure 2:**
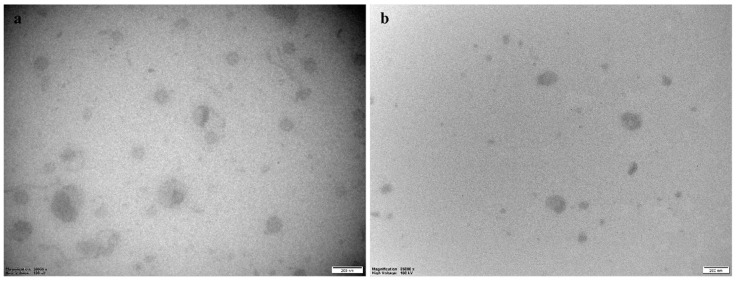
TEM images of chitosomes. (**a**) CHI-EMP, (**b**) CHI-CHX. CHI-EMP = empty chitosomes, CHI-CHX = CHX-chitosomes. Scale bars: 200 nm.

The empty vesicles displayed a slightly smaller size; however, these vesicles served as controls, and the difference would have limited effect on the overall comparison as all vesicles were loaded into hydrogel networks [38]. To confirm the size and to investigate the morphology, we utilized transmission electron microscopy (TEM, Figure 2). Both the empty and CHX-chitosomes were found to be spherical. The size distribution corresponded to the results obtained with the particle sizer. Considering conventional liposomes, the infusion of chitosan did not significantly alter the shape of the vesicles.

The zeta potential of vesicles was highly influenced by both chitosan and CHX (Table 1). Plain, empty vesicles were, due to the high content of phosphatidylcholine, exhibiting neutral surface; the addition of chitosan (chitosomes) augmented the zeta potential by almost 11 mV (Table 1), as expected. The incorporation of CHX in plain vesicles contributed to increased surface charge to 53 mV due to its incorporation within and on the vesicles (Figure 1). The vesicles comprised of both chitosan and CHX (CHX-chitosomes) exhibited the highest zeta potential, indicating that chitosan and CHX have synergic effects on the surface charge. Moreover, these results indicate that both chitosan and CHX are available on the surface of the vesicles or partially stretches out to the surface from within the bilayer. The amphipathic nature of CHX would also substantiate this postulation; however, the substantial increase might suggest that CHX is positioned even further out within the surface of the chitosan-infused vesicles. The zeta potential of plain, empty vesicles and empty chitosomes is directly comparable to the results of Andersen et al. [20]. In topical antimicrobial therapy, positively charged vesicles could be beneficial in bacterial eradication in wounds. Bacterial membranes are slightly negatively charged, whereas mammalian membranes are closer to neutral [40]; therefore, the potential interaction between a positively charged formulation and the bacteria could improve both efficacy and safety [41]. As reported by Ahani and colleagues, where cationic liposomes were proven beneficial in bacterial eradication [42].

The pH of vesicle suspensions was also influenced by CHX presence; an increased pH of more than one unit was determined for CHX-formulations as compared with the corresponding formulation without CHX. Additionally, the effect of acetic acid used in the production of chitosomes was detected in the pH values.

Due to the interactions between CHX and the vesicles and the increased zeta potential, we anticipated a relatively high drug entrapment. However, chitosan could potentially influence the accommodation of CHX within or on the bilayer. High entrapment is important in the development of novel antibacterial formulations to ensure sufficient bacterial eradication and avoid bacteria regrowth. The entrapment efficiencies for both the plain vesicles and chitosomes were relatively high (Table 1). Remarkably, the entrapment was not influenced by the inclusion of chitosan in the vehicles. The high entrapment could also be a result of the interaction between the lipids of the vesicular bilayer and CHX.

#### 2.1.2. Surface-Available Chitosan

The presence of chitosan on the surface of the chitosomes is indicated by the rise of the zeta potential as compared to the plain vesicles. We sought to compare the initial chitosan concentration with the amount available on the chitosome surface. In addition, we investigated whether the concentration of surface-available chitosan would be affected by the incorporation of CHX within the vesicles. The percentage of surface-available chitosan is presented in Table 2. As seen in the table, the surface-available chitosan for chitosomes both with and without CHX was approximately the same. The zeta potential indicates that CHX was positioned within the bilayer; however, the co-accommodation of chitosan was not influenced by the presence of CHX. In antimicrobial therapy, the aim is to preserve chitosan on the surface of the vesicles, allowing chitosan to interact with the bacteria and cause disturbance to the bacterial membrane, since this is considered crucial for its antimicrobial effects [25]. Additionally, we wanted to exploit the potential anti-inflammatory properties of chitosan hydrogel as well as the chitosomes [43]. As indicated in Table 2, approximately 50% of the initial chitosan concentration was present on the vesicle surface, as expected considering the molecular size of chitosan. Moreover, chitosan was accessible to interact with both bacteria and macrophages, therefore improving the healing.

#### 2.1.3. Vesicle Stability

Vesicle stability should be improved upon their incorporation in hydrogel; nevertheless, we evaluated the stability of the vesicle suspensions two and four weeks after preparation to assure that even suspensions are stable (Table 3). The stability of these suspensions is influenced by the zeta potential. Two formulations, namely PL-CHX and CHI-CHX, had a zeta potential above 30 mV, which is expected to stabilize vesicles and preserve their homogeneity [41]. The vesicle size and zeta potential of CHX-loaded formulations did not change significantly (defining significant over 95%) throughout these four weeks, as expected, indicating that the repulsing effects of the CHX-chitosomes and CHX-vesicles are strong enough to stabilize the suspension. However, the empty chitosomes had a significant increase in zeta potential between the second and fourth week (*p* = 0.0005), which would imply that hydrogels are needed to preserve the stability of drug-free chitosomes. In addition, the empty, plain vesicles also exhibited a significant change in the zeta potential between preparation and second week (*p* = 0.009), displaying less stability of these vesicles with surfaces closer to neutral. The pH of all formulations was unaffected during the four weeks of the stability evaluation.

### 2.2. Hydrogel Characterization

#### 2.2.1. Hydrogel Characterization

**Figure 3:**
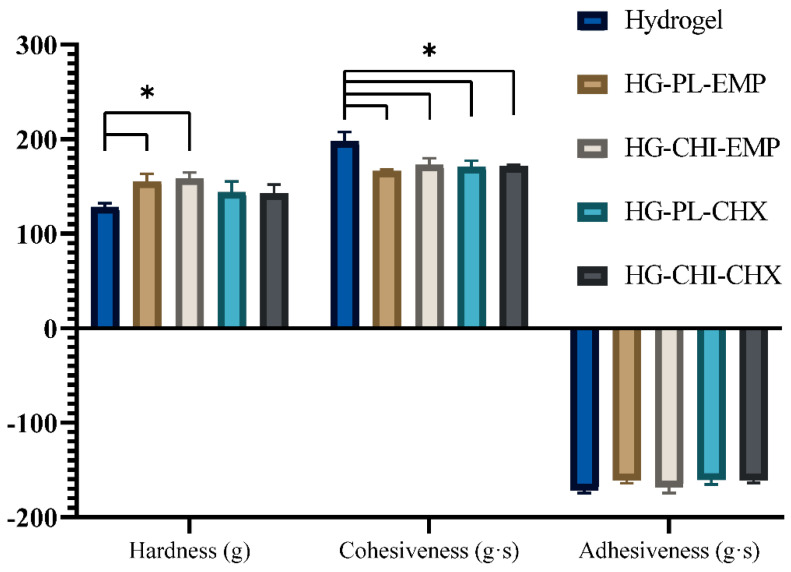
Texture properties of the different chitosan hydrogel formulations All results are expressed as means with their respective SD (*n* = 3). Hydrogel = plain hydrogel, HG-PL-EMP = plain, empty vesicles-in-hydrogel, HG-CHI-EMP = empty chitosomes-in-hydrogel, HG-PL-CHX = plain, CHX-vesicles-in-hydrogel, HG-CHI-CHX = CHX-chitosomes-in-hydrogel. * *p* < 0.05.

Texture analysis is an easy method to monitor the hydrogel production, both as an in-process control as well as a method to determine the effects of modifications in the hydrogel composition [44]. Moreover, it can be utilized for the monitoring of long-term hydrogel stability [45]. Considering the use of hydrogels as skin formulations, this method has been utilized to assess the user-friendliness of both conventional and physical chitosan hydrogels [45,46]. We aimed to utilize the procedure as an in-process control and examine the texture properties upon incorporation of the different vesicles into the original chitosan network. This analysis generates the hardness, cohesiveness, and adhesiveness as quality attributes of the hydrogels. The hardness is expressed as the maximum force required for compressing the hydrogel. The cohesiveness is the level of deformation to the hydrogel upon compression, whereas the adhesiveness describes the hydrogel’s adhesion to the probe compressed into the hydrogel [44]. All parameters for all five hydrogel formulations are presented in Figure 3.

The hardness of the hydrogels incorporating empty vesicles, both plain vesicles and chitosomes, increased compared to the plain (vesicle free) chitosan hydrogel. This increased hardness is in accordance with the findings by Jøraholmen et al. [45]; however, the slight increase in the mean hardness of the CHX-vesicles-containing hydrogels is not significant compared to the plain hydrogel or the hydrogels without CHX. The cohesiveness of the plain chitosan hydrogel was significantly higher than all other formulations (Figure 3). These findings are deviating from our previously reported results on conventional liposomes incorporated in hydrogel. However, the adhesiveness data were in agreement with our previous findings [18]. Moreover, we used texture analysis to determine the stability of the hydrogel formulations; all hydrogels proved to remain relatively stable over a period of four weeks (Table S1).

Considering the pH measurements, no larger variations between the different hydrogels were observed. The values were ranging between the plain hydrogel, with the lowest pH at 4.6, to HG-PL-EMP, displaying the highest pH of 4.9. The rest of the hydrogels had a pH of 4.7. Normal, intact human skin has a pH between 4 and 6 [47], while wounds often display a more alkaline environment [48]. It was suggested that wound healing is improved under more acidic conditions [49], and that the optimal growth conditions of many common skin pathogens are closer to neutral [48]. Therefore, restoring the acidic wound environment would be considered advantageous. Our hydrogels would clearly restore the acidic environment and potentially enhance the healing process. Nevertheless, it is important to state that an acidic pH of skin dressings alone is not sufficient to maintain proper healing cascades [50]. Therefore, we utilized chitosan and CHX to enhance the antimicrobial and anti-inflammatory properties.

#### 2.2.2. Viscosity Evaluation

**Figure 4:**
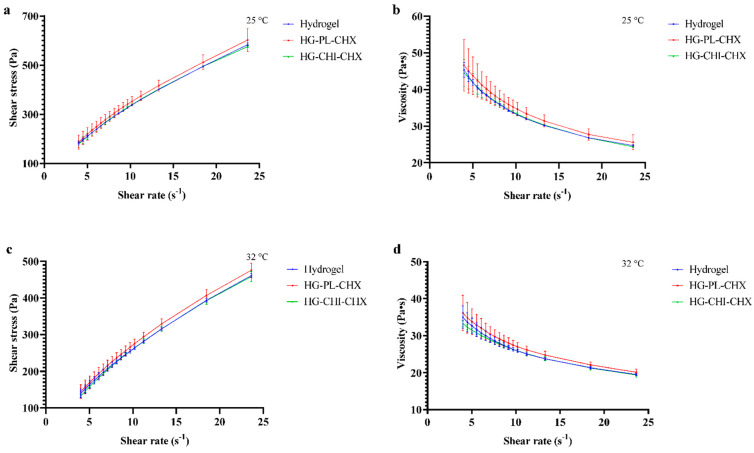
Rheological characteristics. Shear rate was plotted against shear stress (**a**,**c**) and viscosity (**b**,**d**) at 25 °C (**a**,**b**) and 32 °C (**c**,**d**). The results are expressed as means with their respective SD (*n* = 3). Hydrogel = plain hydrogel, HG-PL-CHX = plain, CHX-vesicles-in-hydrogel, HG-CHI-CHX = CHX-chitosomes-in-hydrogel.

In addition to the texture analysis, we sought to investigate the rheological behavior of the plain hydrogel and hydrogels comprising CHX-vesicles. The rheological behavior could elucidate the applicability and therefore the user-friendliness of semi-solid formulations [51]. These properties could be influenced by the temperature. Consequently, we evaluated the hydrogels at 25 °C (Figure 4a,b) and 32 °C (Figure 4c,d), corresponding to dermal application. As seen in Figure 4, the shear stress increased (Figure 4a,c) and viscosity decreased (Figure 4b,d) with increasing shear rate. All hydrogels demonstrated pseudoplastic flow with shear thinning behavior. The rheological behavior was seemingly not influenced by the incorporation of CHX-chitosomes or plain vesicles with CHX. We did not observe any differences in viscosity between different hydrogels as we did for the cohesiveness determined in the texture analysis. Kaplan and colleagues incorporated liposomes in chitosan hydrogel and observed decreased viscosity upon the incorporation of liposomes [52]. However, in their study, the chitosan concentration was significantly lower than in our study. Phospholipids are known to act as plasticizers [53]; therefore, they could increase the mobility within the hydrogel network, leading to a decreased viscosity. Yet, this was not observed in our study. The rheological behavior of vesicles-in-hydrogel is highly influenced by the composition of carriers, lipid concentration, type of polymer, and polymer concentration [54].

Contrary to the effect of incorporation of vesicles into the hydrogel network, the temperature affected the rheological behavior of all hydrogels. The same trends observed at 25 °C were observed at 32 °C as well; however, shear stress and viscosity were significantly lowered at 32 °C. In pharmaceutical formulations, both the shear thinning behavior and the lowered viscosity at application-site temperature (32 °C for skin) could improve the user-friendliness upon administration [51].

### 2.3. CHX Release

**Figure 5:**
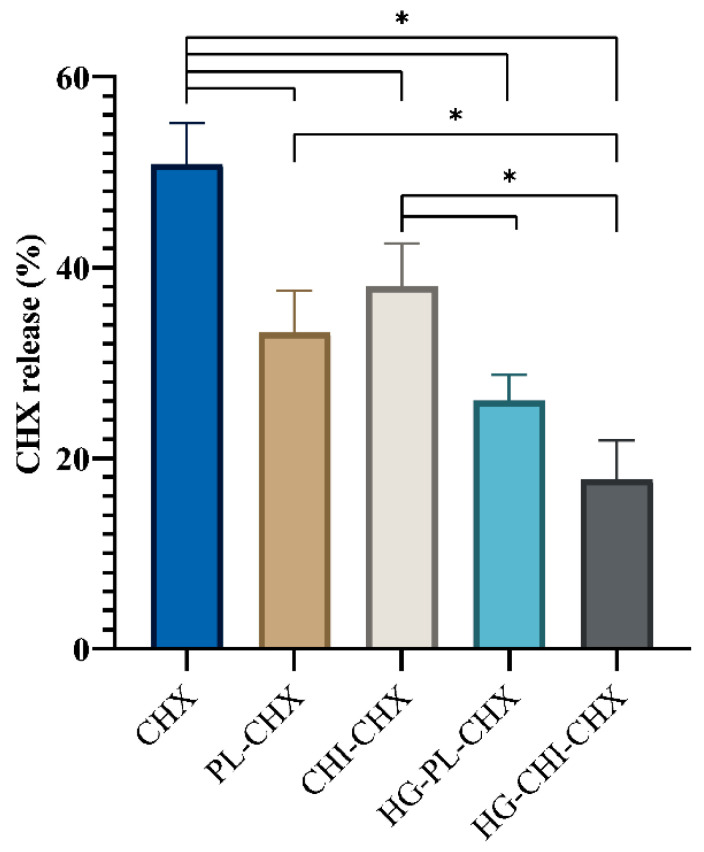
CHX release and permeation from formulated and free CHX after 24 h utilizing the Franz diffusion system (32 °C). The release is presented as the percentage of the initial concentration and all formulations were adjusted to the same initial concentration. All results are expressed as means with their respective SD (*n* = 3). CHX = dissolved CHX, PL-CHX = plain, CHX-vesicles, CHI-CHX = CHX-chitosomes, HG-PL-CHX = plain, CHX-vesicles in hydrogel, HG-CHI-CHX = CHX-chitosomes in hydrogel. * *p* < 0.05.

Topical, localized treatment of burn injuries and acute wounds is preferred, as this provides sufficient concentration of the antimicrobial compound in the infected area [55]. Consequently, patients could avoid both bacterial regrowth and unnecessary adverse systemic effects. We compared the CHX release and permeation from formulated CHX, both the vesicles and vesicles-in-hydrogel, to CHX dissolved in the acceptor medium (Figure 5). As anticipated, the dissolved CHX permeated faster than CHX from all other formulations. Only the CHX-chitosomes released a significantly greater amount than both vesicles-in-hydrogel formulations under the tested conditions. The CHX-chitosomes seemingly had a higher mean release than the plain vesicles with CHX. This might be due to the competition between CHX and chitosan within the lipid bilayer of the vesicles, as CHX might be expelled. Interestingly, comparing the vesicles-in-hydrogel, the CHX release from the formulation comprising chitosomes displayed sustained release; however, it was not significantly relevant. We postulate that this effect might be due to the effect of the positive charge of the surrounding chitosan hydrogel network. The zeta potential of CHX-chitosomes was significantly higher than the zeta potential of plain vesicles with CHX (Table 1), which might lead to stronger repulsion between the hydrogel and the CHX-chitosomes. This similar effect has previously been demonstrated by Hurler and colleagues [38]. This repulsive effect could also stabilize the vesicles incorporated in the hydrogel network. However, the effect of the wound exudate should not be neglected [18]. Moreover, in an in vivo challenge, the hydrogel would be exposed to wound bed comprising exudates and blood components resulting in its swelling [30].

Vesicles-in-hydrogels often offer a prolonged drug release profile, important for chronic wound treatment [56].

### 2.4. Evaluation of Potential Toxicity

**Figure 6:**
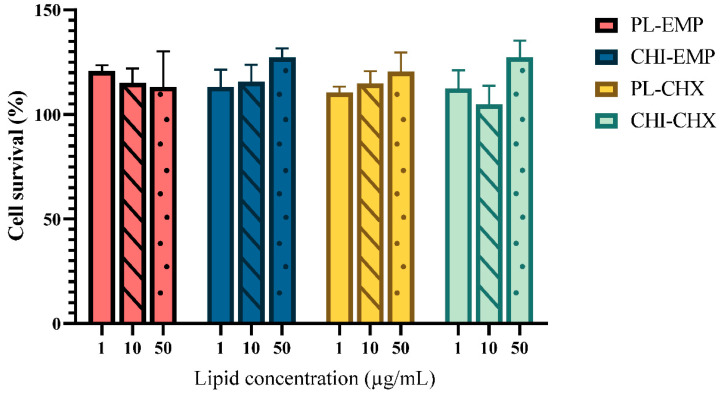
Evaluation of vesicles cell toxicity in HaCaT cells. Three different concentrations were tested, namely 1 (no pattern), 10 (stripes), and 50 (dots) µg/mL lipid, and the results are presented as cell viability of treated cells compared to control (100%). Control was only supplemented with complete medium; the cell viability is thereof considered as 100%. All results are expressed as means with their respective SD (*n* = 3). PL-EMP = plain, empty vesicles, CHI-EMP = empty chitosomes, PL-CHX = plain, CHX-vesicles, CHI-CHX = CHX-chitosomes.

**Figure 7:**
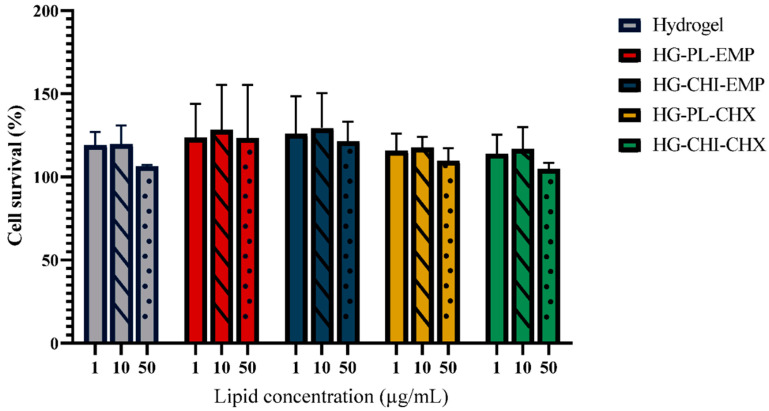
Evaluation of cell toxicity of hydrogels on HaCaT cells. Three different concentrations were tested, namely 1 (no pattern), 10 (stripes), and 50 (dots) µg/mL lipid (or the corresponding chitosan concentration), and the results are presented as cell viability of treated cells compared to control (100%). Control was only supplemented with complete medium; the cell viability is thereof considered as 100%. All results are expressed as means with their respective SD (*n* = 3). Hydrogel = plain hydrogel, HG-PL-EMP = plain, empty vesicles-in-hydrogel, HG-CHI-EMP = empty chitosomes-in-hydrogel, HG-PL-CHX = plain, CHX-vesicles-in-hydrogel, HG-CHI-CHX = CHX-chitosomes-in-hydrogel.

The biocompatibility of any formulation intended for burns and other wounds is essential for a successful treatment outcome. Reduced cell compatibility could prevent or delay the intricate healing cascade. After skin disruption, keratinocytes migrate and proliferate to close the wound area and are, together with fibroblasts, fundamental in the healing process [57]. Therefore, cell toxicity studies were performed for both vesicles (Figure 6) and hydrogels (Figure 7) after 24 h exposure of each formulation to keratinocytes. The treated cells were compared with non-treated cells to assess the safety and compatibility of each formulation. As seen in Figure 6, the vesicles did not impair the cell survival, regardless of their concentration. Additionally, the highest lipid concentration (50 µg/mL) of chitosomes exhibited a significantly improved cell proliferation as compared to the cells treated with only medium (control). Both empty chitosomes (*p* = 0.02) and CHX-chitosomes (*p* = 0.01) improved cell survival in the highest lipid concentration. The improved proliferation of keratinocytes exposed to chitosan can be attributed to its positive effects on cell growth. The vesicles and chitosomes with CHX appeared to display a concentration-dependent trend with improved cell viability in the highest concentrations. Other chitosan-comprising formulations such as chitosan-coated liposomes have been evaluated in various cell lines. Mengoni and colleagues demonstrated compatible chitosan-coated liposomes in keratinocytes (HaCaT cells) [58]. Phetdee and colleagues investigated the proliferation in HaCaT cells treated with chitosan-coated liposomes and reported no negative proliferative effects [59]. Additionally, proliferative effects have been reported in fibroblasts treated with chitosan [60]. On the other hand, CHX has been shown to demonstrate toxicity in both fibroblasts [61] and keratinocytes [62]; however, we did not detect any toxicity issues with CHX-chitosomes (Figure 6).

In addition to the evaluation of the vesicles compatibility, we investigated the cell compatibility of hydrogels (Figure 7). The hydrogels did not exhibit any toxicity toward the keratinocytes; however, none of the hydrogels significantly improved cell survival. The cell compatibility of hydrogels or other wound dressing materials has previously been reported in both keratinocytes and fibroblasts [63,64,65]. Additionally, Hurler and colleagues demonstrated in a murine burn model that liposomes-in-hydrogel formulations with mupirocin were safe [66]. Chitosan is generally regarded as both safe and biocompatible [12]. However, the degree of deacetylation and chitosan concentration play an important role in cell compatibility. Due to the complex process of wound healing, the full extent of the underlying mechanisms responsible for the effects of chitosan on keratinocytes or fibroblasts are not fully elucidated [60,67]. However, chitosan appears to support granulation and remodeling through its effects on the inflammatory cells and growth factors [68]. Certain growth factors are important in the migration and proliferation of keratinocytes [69]. Consequently, the effects of chitosan-based formulations on inflammatory cells are important to monitor.

In the inflammation phase, immune cells are requited to the wound bed, and some cells differentiate into macrophages. These cells initiate a process that coordinates other cells in the overlapping phases in the healing process as well as combats microorganisms in the injured area [70]. The involvement of macrophages in the wound-healing cascade is extensive and not fully elucidated [71]. We have previously confirmed a decreased inflammatory activity in cells treated with chitosan formulations [18]. The CHX-chitosomes have not been evaluated for their potential effect on macrophages earlier. Figure S1 indicates that chitosan-infused vesicles did not potentiate immune response. Interestingly, the plain vesicles demonstrated a dose-dependent reduction of the inflammatory response in lipopolysaccharide (LPS)-induced murine macrophages compared to untreated activated cells. These results are promising considering application in wound therapy.

In the wound-healing process, both cell compatibility and inflammatory responses are important factors. Additionally, the ability of cells to migrate into the wound bed to close the wound area is equally important for the wound-healing process. The impact of chitosan on the migratory abilities of different cell lines was previously evaluated [35], and the results are encouraging for our system. Formulations containing chitosan have demonstrated improved cell migration in fibroblasts [72], macrophages [73], and keratinocytes [74].

### 2.5. Antimicrobial Evaluation

Tailoring drug delivery systems comprising chitosan to optimize its intrinsic antimicrobial activity could improve the effect of the formulation itself [14]. Chitosan is known to act against *Staphylococcus aureus*, which is one of the most common skin pathogens [75] as previously reported [76,77]. Although the mechanisms of the antimicrobial activity of chitosan are not fully elucidated, the electrostatic interaction between the slightly negatively charged bacterial membrane and the positively charged chitosan groups is the most common explanation [76]. In addition, reports suggest that chitosan, especially higher molecular weight chitosan, could form an envelope around the bacteria, depriving them of nutrients and closing of the exchange with the surrounding environment [78]. These strong effects on the bacteria could act in synergy with MAAs such as CHX. Therefore, we sought to compare plain vesicles and chitosomes both with and without CHX to assess the potential antimicrobial effects. Through the modified broth dilution method, we demonstrated a lowered minimum bactericidal concentration (MBC) in both *S. aureus* and *Staphylococcus epidermidis* cultures from formulations comprising both CHX and chitosan compared to their respective controls (Table 4). Chitosomes without CHX and CHX-vesicles displayed improved activity compared to the plain, empty vesicles. As expected, plain, empty vesicles did not eradicate a sufficient number of bacteria to reach MBC, neither with *S. aureus* nor *S. epidermidis*. However, in the highest concentration, the plain-empty vesicles reduced the *S. epidermidis* colony count by approximately 50%. The antimicrobial activity of CHX-chitosomes against both bacteria was proven to be superior to the other vesicles, indicating that there is a synergetic effect between CHX, our model MAA, and chitosan, as hypothesized.

Alshamsan and colleagues evaluated the antibacterial efficacy of chitosan-coated and non-coated liposomes loaded with dicloxacillin against methicillin-resistant *S. aureus*. Dicloxacillin, commonly used in skin infections, demonstrated improved activity of non-coated liposomes; however, the activity of coated liposomes was retained compared to dicloxacillin in solution [79]. Chitosan-coated liposomes have also demonstrated promising antimicrobial effects in colistin-resistant *Pseudomonas aeruginosa* [80]. Sacco and colleagues evaluated a physical chitosan hydrogel against *S. epidermidis* and revealed promising antimicrobial activity [81]. These results along with other reports [82] demonstrate the promising antimicrobial effects of chitosan-coated or infused vesicles in antimicrobial treatment.

Since secondary vehicles are required in wound therapy, we aimed to investigate whether chitosan hydrogel could further improve the effect of chitosan-infused vesicles with CHX. Jøraholmen and colleagues compared the antimicrobial effects of both chitosan hydrogel and chitosan-coated liposomes against both *S. aureus* and *S. epidermidis* and reported promising effects of chitosan in low concentrations [14]. As seen in Table 5, for *S. aureus*, almost all hydrogels exhibited a similar antimicrobial effect; only the CHX-chitosomes-in-hydrogel showed slightly lowered MBC compared to the other hydrogel formulations. However, the MBC for all hydrogels was lowered as compared to the vesicular suspensions. For *S. epidermidis*, the effects of different vesicles incorporated in the hydrogel were more evident (Table 5). The activity increased upon the addition of CHX, chitosan, and their combination. The most potent formulation was CHX-chitosomes-in-hydrogel. Moreover, these results indicate that even a diluted hydrogel with a modified chitosan network structure acts on improving the antimicrobial activity. The findings confirmed that vesicle surface-available chitosan in combination with CHX induces the strongest activity also when those vesicles were arranged within a chitosan network.

## 3. Materials and Methods

### 3.1. Materials

Chitopharm™ M-Chitosan with medium molecular weight (average of 350–600 kDa) and degree of deacetylation of >70% from shrimp was kindly provided by Chitinor (Tromsø, Norway). Lipoid S100 was kindly provided by Lipoid GmbH (Ludwigshafen, Germany). Methanol ≥ 99.9%, HiPerSolv CHROMANORM^®^ for LC-MS and acetic acid (>99.9%) were purchased from VWR International (Fontenay-sous-Bois, France). Cibacron Brilliant Red 3B-A was procured from Santa Cruz Biotechnology (Dallas, TX, USA). Chlorhexidine > 99.5%, glycerol solution (86–89%), glycine hydrochloride ≥ 99% (HPLC), sodium chloride, hydrochloric acid, Cell Counting Kit-8 (CCK-8), and Kollisolv^®^ PEG E 400 were acquired from Sigma-Aldrich (St. Louis, MO, USA). 1-Propanol, penicillin–streptomycin, and fetal bovine serum (FBS) were purchased from Sigma-Aldrich (Steinheim, Germany). Blood agar plates, saline solution, and Mueller–Hinton broth were delivered by University Hospital of North Norway (Tromsø, Norway). Dulbecco’s Modified Eagle Medium high glucose (DMEM HG) w/l-glutamine and sodium pyruvate was purchased from Biowest (Nuaillé, France). HaCaT cell line (immortalized human keratinocytes) was purchased from CLS Cell Lines Service GmbH (Eppelheim, Germany). *Staphylococcus aureus* (ATCC^®^ BAA-1721™) MSSA 476 was purchased from LGC standards AB (Borås, Sweden). *Staphylococcus epidermidis* (13–67) was delivered by University Hospital of Northern Norway (Tromsø, Norway).

### 3.2. Vesicle Preparation

#### 3.2.1. Vesicle Preparation

The preparation of chitosomes was based on the one-pot method previously described by Andersen et al. [31]. In short, Lipoid S100 (200 mg) and CHX (10 mg) were dissolved in methanol and a lipid film was formed by evaporation of the solvent in a rotoevaporator (Büchi rotavapor R-124, with vacuum controller B-721, Büchi vac V-500, Büchi Labortechnik, Flawil, Switzerland) at 60 mBar and 45 °C for 1 h. A micro syringe (Innovative Labor Systeme GmBH, Stutzerbach, Germany) filled with 150 µL 1-propanol was used to disperse the lipid film. The 1-propanol/lipid dispersion was further injected into a chitosan dispersion (0.17%, *w*/*w*, 2 mL) in acetic acid (0.1%, *v*/*v*) under continuous mechanical stirring. Finally, the resulting suspension was stirred for another 2 h at room temperature (24 ± 1 °C) and stored in the refrigerator (4 °C) prior to size reduction. Formulations without chitosan were prepared in the same manner; however, the 1-propanol/lipid dispersion was injected into distilled water (2 mL) instead of the chitosan dispersion. Formulations without CHX was prepared in the same way but without CHX. All vesicle designations and constituents are included in Table 6.

#### 3.2.2. Size Reduction

Prior to size reduction, all vesicle suspensions were diluted with distilled water to a lipid concentration of 20 mg/mL. The samples were probe sonicated (SONICS high-intensity ultrasonic processor, 500-watt model, 13 mm probe diameter, Sonics & Materials Inc., Newtown, CT, USA) at 40% amplitude for 10 s and ten times 10 s for the CHX-containing and the empty vesicles, respectively. The sample containers were placed in an ice bath throughout the sonication to avoid extensive heating.

### 3.3. Characterization of Chitosomes

#### 3.3.1. Vesicle Size and Morphology

The size of vesicles was measured on a NICOMP Submicron particle sizer model 370 (NICOMP Particle Sizing system, Santa Barbara, CA, USA) described elsewhere [14]. The suspensions were diluted in filtered (0.2 µm) distilled water to reach an intensity of 250–350 KHz and measured for three cycles of 10 min. The scattering angle of every measurement was 90°, and the temperature was 24 ± 1 °C. The results are expressed as the weight-intensity distribution.

Prior to the morphological investigations, empty chitosomes and CHX-chitosomes were deposited onto carbon-coated grids for 5 min, washed with double-distilled water, and stained with 3% uranyl acetate and 2% methylcellulose (1:9) for 2 min. The samples were picked up with a loop and dried on the loop holder. The images were obtained with a transmission electron microscope HT7800 Series (Hitachi High-Tech Corp., Tokyo, Japan) operating at an accelerated voltage of 100 kV coupled with a Morada camera.

#### 3.3.2. Zeta Potential and pH of the Vesicles

The zeta potential was determined with a Malvern Zetasizer Nano Zen 2600 (Malvern, Worcestershire, UK) as described earlier [83]. Zeta cells were rinsed three times with methanol and filtered, deionized water prior to the measurements. The suspensions were measured in three replicates at room temperature (24 ± 1 °C).

Determination of the pH was carried out with an Accumet^®^, Portable pH meter AP115 (Fischer Scientific, MA, USA) at room temperature (24 ± 1 °C).

#### 3.3.3. Separation and Entrapment Efficiency

The free CHX was separated from the entrapped CHX by centrifugation [84]. The chitosomes were centrifuged at 4000× *g* and 4 °C for 30 min on the Biofuge Stratos centrifuge (Heraeus Instruments GmbH, Hanau, Germany). Entrapment analysis was carried out on the SPARK^®^ multimode microplate reader (Tecan Trading AG, Männedorf, Switzerland) at 261 nm.

#### 3.3.4. Determination of Availability of Chitosan on the Surface

The determination of surface-available chitosan was based on a method described by Muzzarelli [85]. Prior to the determination, the chitosomes were centrifuged in a centrifugal filter (Amicon Ultra-2 Centrifugal Filter Unit Ultracel-10, Sigma-Aldrich, St. Louis, MI, USA) at 3118× *g* for 15 min on the Biofuge Stratos centrifuge (Heraeus Instruments GmbH, Hanau, Germany) [86]. First, glycine and NaCl was dissolved in distilled water in concentrations of 0.748% (*w*/*v*) and 0.584% (*w*/*v*), respectively. A glycine buffer with pH 3.2 was prepared by diluting 81 mL of the glycine and NaCl solution with 0.1 M HCl to a total volume of 100 mL. Next, a dye solution was prepared by dissolving Cibacron Brilliant Red 3B-A (0.15%, *w*/*v*) in distilled water and 5 mL of this solution was diluted in glycine buffer to a total volume of 100 mL. The centrifuged chitosomes were diluted (1:1, *v*/*v*) in distilled water. An aliquot of 3 mL of the dye solution was added to 300 µL of the diluted chitosomes, and the samples were analyzed on a UV-vis plate reader (Tecan Trading AG, Männedorf, Switzerland) at 575 nm [87].

#### 3.3.5. Chitosome and Vesicle Stability

The physical properties of chitosomes and plain vesicles (stored at 4 °C) were evaluated after storage for two and four weeks after preparation. Properties evaluated were size, PI, zeta potential, and pH as described in Section 3.3.1 and Section 3.3.2.

### 3.4. Preparation and Characterization of Hydrogels

#### 3.4.1. Preparation of Chitosan Hydrogel

Chitosan hydrogels comprising glycerol as a plasticizer were prepared in 2.5% (*w*/*w*) acetic acid in distilled water. The dispersions were mixed with a Cito Unguator^®^ 2000 (GAKO International AG, Zurich, Switzerland) and degassed by bath sonication (Bransonic^®^ 5510R-MT Ultrasonic cleaner, Branson Ultrasonics Corporation, Danbury, CT, USA) for 30 min. The final concentrations of chitosan and glycerol were 4.5 and 9%, respectively. Hydrogels were allowed to swell for 48 h prior to characterization or the incorporation of vesicles.

The vesicles-in-hydrogel were prepared by incorporating 10% (*w*/*w*) vesicle suspension into chitosan hydrogels of 5% chitosan and 10% glycerol, respectively, by hand-stirring for 5 min. The concentration of chitosan and glycerol after the incorporation of vesicular suspensions were 4.5 and 9%, respectively. All hydrogel designations and their composition are included in Table 7.

#### 3.4.2. Texture Properties and pH of Hydrogels

Texture properties of hydrogels were evaluated on the TA.XT plus Texture Analyser (Stable Micro Systems Ltd., Surrey, UK) with a backward extrusion rig as previously described by Hurler et al. [44]. The beaker of the rig set was filled with 65 g hydrogel and the disc (35 mm) was compressed into the hydrogel and withdrawn back to the starting position (above the surface). The measuring distance was 10 mm and the trigger force was set to 10 g. The pre-test, test, and post-test speeds were 10, 4, and 4 mm/s, respectively. Hardness, cohesiveness, and adhesiveness were recorded.

The pH of all hydrogels were measured with an Accumet^®^, Portable pH meter, AP115 (Fisher scientific, Waltham, MA, USA) at room temperature (24 ± 1 °C).

#### 3.4.3. Viscosity Measurements

The measurements of viscosity were performed on a Rotavisc hi-vi II Complete coupled with DINS-1 adapter with spindle DIN-SP-7 and DIN-C-2 chamber (IKA^®^-Werke GmbH & Co. KG, Staufen, Germany). Both viscosity and shear stress was evaluated as a function of the shear rate [51]. The shear rate range was between 4.0 s^−1^ and 23.63 s^−1^ and the temperature was set to 25 or 32 °C.

### 3.5. CHX Release Studies

CHX release was determined in a Franz cell diffusion system (PermeGear, Hellertown, PA, USA) with circulating heated water of 32 °C. The diffusion area of the pre-soaked cellophane membrane (Max Bringmann KG, Wendelstein, Germany) was 1.77 cm^2^ and the acceptor volume was 12 mL. Due to the lowered water solubility of CHX, the acceptor chamber was filled with polyethylene glycol 400 (10%, *v*/*v*) in distilled water. The formulations (600 µL) were added to the donor chamber. Samples were withdrawn from the donor chamber after 24 h and analyzed as described in Section 3.3.3. The formulations were compared with free CHX dissolved in the acceptor medium (permeation). The donor chamber was weighed before and after every run to adjust for fluid exchange, and therefore, the samples were measured only after 24 h [18].

### 3.6. Cell Viability Valuation

The cytotoxicity of formulations was evaluated using a Cell counting kit–8 (CCK-8, Sigma-Aldrich Chemie, St. Louise, MI, USA) as described elsewhere [88]. Briefly, an aliquot of 90 µL cell suspension cultured in DMEM HG supplemented with 10% (*v*/*v*) FBS and 1% (*v*/*v*) penicillin–streptomycin (1 × 10^5^ cells/mL) were plated on a 96-well plate and incubated for 24 h at 37 °C with 5% CO_2_. Next, 10 µL of medium (control), diluted vesicle suspension, or diluted hydrogel (1, 10, and 50 µg/mL lipid concentration or the corresponding concentration of hydrogels) was added to the wells. The cells were incubated for another 24 h at 37 °C with 5% CO_2_. After incubation, 10 µL CCK-8 was added to each well, and the plates were incubated for 4 h. Finally, the plates were evaluated at a UV-vis microplate reader (Tecan Trading AG, Männedorf, Switzerland) at 450 nm with the reference set to 650 nm. All formulations were evaluated in triplicates and the results were expressed as percentage compared to control.

### 3.7. Antimicrobial Evaluation

In the microbial evaluation, we sought to calculate the MBC for each formulation to compare the effect of every modification for both vesicles and hydrogels. Here, we used a modified broth micro-dilution method [89,90]. Two species were evaluated, namely *S. aureus* MSSA 476 and *S. epidermidis* (13–67). Prior to the experiments, all hydrogels were diluted 1:4 (*v*/*v*) in distilled water. All formulations were two-fold diluted in Mueller–Hinton broth in sterile 96-well plates. Bacterial suspensions were prepared at 0.5 McFarland in 0.85% (*w*/*w*) sodium chloride solutions, corresponding to approximately 10^8^ CFU/mL. The bacterial suspensions were further diluted (1:150, *v*/*v*) in Mueller–Hinton broth. The inoculum was added to each well (1:1, *v*/*v*) in the 96-well plate and incubated at 37 °C on a shaker (100 rpm) for 24 h. The wells with only bacteria and Mueller–Hinton broth served as positive and negative controls, respectively. After 24 h incubation, the bacterial suspensions were 10-fold serial diluted in phosphate-buffered saline, plated on blood agar plates, and incubated at 37 °C overnight [90]. The CFUs of the bacteria treated with formulations were compared to the control (only growth medium) and the MBC (lipid concentration) was determined.

### 3.8. Statistical Analyses

In general, results are expressed as mean ± SD. Student’s t-tests or one-way ANOVA with Tukey post-test were performed to evaluate significance (*p* < 0.05). All statistical analyses were performed in GraphPad Prism version 9.0.0 for Windows (GraphPad Software LLC, San Diego, CA, USA).

## 4. Conclusions

Novel formulations for prevention and treatment of acute skin injuries prone to infections are highly needed. This study supported the hypothesis that chitosan-infused lipid-based vesicles, chitosomes loaded with CHX and incorporated into chitosan hydrogel network could serve as a suitable formulation for infection control, prevention, and eradication of bacterial infections in acute wounds. The novel formulation displayed safety and superior antimicrobial properties, which are both highly desirable for topical therapy of infected wounds. Additionally, the combination of chitosan and CHX could provide both a faster onset of the antimicrobial action and additionally offer a long-term effect on bacteria in wounds.

## Figures and Tables

**Table 1:** Vesicle characteristics.

	Size (nm)			PI ^1^	Zeta Potential (mV)	EE ^2^ %	pH
	Peak 1 %	Peak 2 %	Peak 3 %				
PL-EMP	31 ± 9 5 ± 3	62 13	169 ± 18 90 ± 4	0.18 ± 0.01	0.6 ± 0.0	-	5.6 ± 0.0
CHI-EMP	14 ± 4 5 ± 4	41 ± 4 30 ± 12	150 ± 3 65 ± 16	0.22 ± 0.01	11.5 ± 0.3	-	4.4 ± 0.0
PL-CHX	16 ± 7 2 ± 1	66 ± 15 16 ± 5	243 ± 13 81 ± 6	0.32 ± 0.03	53.6 ± 2.0	68 ± 5	7.0 ± 0.3
CHI-CHX	14 ± 1 3 ± 1	79 ± 5 29 ± 15	260 ± 3 69 ± 16	0.30 ± 0.00	79.0 ± 3.7	74 ± 2	5.5 ± 0.1

Results are expressed as means with their respective SD (*n* = 3). PL-EMP = plain, empty vesicles, CHI-EMP = empty chitosomes, PL-CHX = plain, CHX-vesicles, CHI-CHX = CHX-chitosomes. ^1^ Polydispersity index. ^2^ Entrapment efficiency (%).

**Table 2:** Surface-available chitosan of the empty and loaded chitosan-infused vesicles.

	Surface-Available Chitosan (%) ^3^
CHI-EMP	50.2 ± 2.9
CHI-CHX	48.5 ± 5.6

Results are expressed as means with their respective SD (*n* = 3). CHI-EMP = empty chitosomes, CHI-CHX = CHX-chitosomes. ^3^ Percentage of initial chitosan concentration (%).

**Table 3:** Surface-available chitosan on the empty and CHX-loaded chitosomes.

	Week	Size (nm)			PI ^1^	Zeta Potential (mV)	pH
		Peak 1 %	Peak 2 %	Peak 3 %			
PL-EMP	2	33 ± 3 6 ± 2	133 ± 36 72 ± 35	331 ± 246 33 ± 39	0.20 ± 0.02	−1.7 ± 0.4	5.6 ± 0.1
	4	17 ± 1 2 ± 1	69 ± 21 26 ± 27	229 ± 49 62 ± 31	0.21 ± 0.02	−3.1 ± 1.0	5.6 ± 0.4
CHI-EMP	2	18 ± 2 3 ± 1	58 ± 9 15 ± 2	152 ± 3 82 ± 1	0.22 ± 0.01	12.0 ± 0.2	4.4 ± 0.0
	4	18 ± 5 4 ± 1	56 ± 6 23 ± 23	144 ± 24 86 ± 1	0.22 ± 0.01	14.4 ± 0.5	4.5 ± 0.1
PL-CHX	2	11 ± 0 1 ± 1	64 ± 8 18 ± 4	254 ± 21 81 ± 4	0.33 ± 0.03	55.9 ± 0.9	6.9 ± 0.2
	4	22 ± 13 4 ± 3	101 ± 76 43 ± 43	225 ± 11 82 ± 5	0.32 ± 0.03	55.7 ± 1.0	7.2 ± 0.1
CHI-CHX	2	14 ± 3 3 ± 3	54 ± 9 21 ± 20	222 ± 41 75 ± 22	0.30 ± 0.01	79.8 ± 4.5	5.5 ± 0.1
	4	12 ± 1 2 ± 1	64 ± 17 21 ± 7	215 ± 49 76 ± 8	0.30 ± 0.02	83.0 ± 1.7	5.5 ± 0.1

Vesicle characteristics evaluated 2 and 4 weeks after preparation. Results are expressed as means with their respective SD (*n* = 3). PL-EMP = plain, empty vesicles, CHI-EMP = empty chitosomes, PL-CHX = plain, CHX-vesicles, CHI-CHX = CHX-chitosomes. ^1^ Polydispersity index.

**Table 4:** MBC of vesicles in *S. aureus* and *S. epidermidis*.

	Lipid Concentration (mg/mL) *S. aureus*	Lipid Concentration (mg/mL) *S. epidermidis*
PL-EMP	-	-
CHI-EMP	1.25	0.625
PL-CHX	0.32	0.039
CHI-CHX	0.078	<0.005

All results are expressed as the lipid concentration upon reaching MBC (*n* = 3). PL-EMP = plain, empty vesicles, CHI-EMP = empty chitosomes, PL-CHX = plain, CHX-vesicles, CHI-CHX = CHX-chitosomes.

**Table 5:** MBC of vesicles in *S. aureus* and *S. epidermidis*.

	Lipid Concentration (mg/mL) ^4^ *S. aureus*	Lipid Concentration (mg/mL) ^4^ *S. epidermidis*
Hydrogel	1.56 × 10^−2^	0.10 × 10^−2^
HG-PL-EMP	1.56 × 10^−2^	0.10 × 10^−2^
HG-CHI-EMP	1.56 × 10^−2^	0.025 × 10^−2^
HG-PL-CHX	1.56 × 10^−2^	0.0063 × 10^−2^
HG-CHI-CHX	0.78 × 10^−2^	0.0031 × 10^−2^

All results are expressed as the lipid concentration upon reaching MBC (*n* = 3). Hydrogel = plain hydrogel, HG-PL-EMP = plain, empty vesicles-in-hydrogel, HG-CHI-EMP = empty chitosomes-in-hydrogel, HG-PL-CHX = plain, CHX-vesicles-in-hydrogel, HG-CHI-CHX = CHX-chitosomes-in-hydrogel. ^4^ Lipid concentration or the corresponding concentration of hydrogel.

**Table 6:** Designation and constituents of all vesicles.

	Composition
PL-EMP	Lipoid S100
CHI-EMP	Lipoid S100 Chitosan
PL-CHX	Lipoid S100 CHX
CHI-CHX	Lipoid S100 Chitosan CHX

**Table 7:** Designation and constituents of all hydrogels.

	Composition
Hydrogel	Chitosan Glycerol
HG-PL-EMP	Chitosan Glycerol PL-EMP vesicles
HG-CHI-EMP	Chitosan Glycerol CHI-EMP vesicles
HG-PL-CHX	Chitosan Glycerol PL-CHX vesicles
HG-CHI-CHX	Chitosan Glycerol CHI-CHX vesicles

## Data Availability

Data is contained within the article and supplementary material.

## Supplementary Materials

The following are available online at https://www.mdpi.com/article/10.3390/md19050269/s1, Table S1: Stability hydrogel measured as texture properties and pH. Figure S1: Evaluation of anti-inflammatory activity of vesicles on RAW 264.7 cells.
