# Supplementary material for: Chitosomes-In-Chitosan Hydrogel for Acute Skin Injuries: Prevention and Infection Control
Source: Mar Drugs. 2021 May 12;19(5):269. doi: 10.3390/md19050269 (PMC8150996; doi:10.3390/md19050269)
Supplement: Supplementary file 1 [file marinedrugs-19-00269-s001.zip › marinedrugs-1211569-supplementary.pdf]

Supplementary material

Chitosomes-in-chitosan hydrogel for acute skin injuries: prevention and infection control

Lisa Myrseth Hemmingsen [1], Kjersti Julin [2], Luqman Ahsan [1], Purusotam Basnet [3,4], Mona Johannessen [2] and Nataša Škalko-Basnet [1*]

[1] Drug Transport and Delivery Research Group, Department of Pharmacy, University of Tromsø The Arctic University of Norway, Universitetsvegen 57, 9037 Tromsø, Norway; lisa.m.hemmingsen@uit.no (L.M.H), luqmaan.ahsan@gmail.com (L.A.), natasa.skalko-basnet@uit.no (N.Š-B)
[2] Research Group for Host-Microbe Interaction, Department of Medical Biology, University of Tromsø The Arctic University of Norway, Sykehusvegen 44, 9037 Tromsø, Norway; kjersti.julin@uit.no (K.J), mona.johannessen@uit.no (M.J)
[3] IVF Clinic, Department of Obstetrics and Gynecology, University Hospital of North Norway, Sykehusvegen 38, 9019 Tromsø, Norway; purusotam.basnet@uit.no (P.B)
[4] Women's Health and Perinatology Research Group, Department of Clinical Medicine, University of Tromsø The Arctic University of Norway, Universitetsvegen 57, 9037 Tromsø, Norway; purusotam.basnet@uit.no (P.B)
* Correspondence: natasa.skalko-basnet@uit.no; Tel.: +47 77646640

S1. Supplementary results

S1.1. Hydrogel stability

The stability of the hydrogels both with and without vesicles was evaluated as the texture properties and pH over time. The texture properties of hydrogels with vesicles demonstrated higher stability over a period of four weeks compared to hydrogel without vesicles (Table S1). The parameters cohesiveness and adhesiveness of the plain hydrogel demonstrated significant changes. This might be attributed to the effects of phospholipids. Since phospholipids could act as plasticizers, which could provide an improved long-term effect. Glycerol was added as a plasticizer in all hydrogels, both to improve the mobility in the hydrogel networks and to improve the stability of the hydrogels [44]. The increased mobility in the hydrogels with vesicles was evident from the lowered cohesiveness in these hydrogels compared to the hydrogel without vesicles. Jøraholmen and colleagues did not observe this effect upon addition of liposomes in a concentration of 10 or 20% in chitosan hydrogel [45]. Additionally, Hurler and colleagues suggested that vesicles with positive zeta potential stabilize the chitosan hydrogel network better than neutral vesicles [38], probably due to the repulsive effects.

The pH of all hydrogels proved stable over the whole period and no significant changes were observed (Table S1).

Table S1. Stability hydrogel measured as texture properties and pH.

	Week	Hardness<br>g	Cohesiveness<br>g·s	Adhesiveness<br>g·s	pH
Hydrogel	2	143 ± 3	177 ± 2	-164 ± 2	4.6 ± 0.0
	4	144 ± 5	190 ± 5	-174 ± 5	4.6 ± 0.0
HG-PL-EMP	2	153 ± 6	160 ± 5	-155 ± 3	4.9 ± 0.1
	4	164 ± 10	166 ± 8	-162 ± 7	4.9 ± 0.1
HG-CHI-EMP	2	160 ± 9	162 ± 2	-159 ± 3	4.8 ± 0.0
	4	165 ± 11	166 ± 5	-162 ± 6	4.8 ± 0.0

HG-PL-CHX	2	139 ± 6	169 ± 10	-156 ± 9	4.7 ± 0.0
	4	141 ± 3	161 ± 5	-152 ± 2	4.7 ± 0.0
HG-CHI-CHX	2	145 ± 8	169 ± 4	-160 ± 4	4.7 ± 0.0
	4	150 ± 6	174 ± 5	-165 ± 3	4.7 ± 0.0

Hydrogel characteristics were evaluated 2 and 4 weeks after preparation. Hydrogel = plain hydrogel, HG-PL-EMP = plain, empty vesicles in hydrogel, HG-CHI-EMP = empty chitosomes in hydrogel, HG-PL-CHX = plain, CHX-vesicles in hydrogel, HG-CHI-CHX = CHX-chitosomes in hydrogel. Results are expressed as means with their respective SD (n = 3).

S1.2. Anti-inflammatory activity

In Figure S1, the inflammatory responses of both chitosomes and plain vesicles with and without CHX are presented. The plain vesicles demonstrated dose-dependent reduction of the inflammation response in lipopolysaccharide (LPS)-induced murine macrophages compared to untreated activated cells, whereas the chitosan-infused vesicles maintained the same response as the non-treated cells and did not introduce any additional inflammation response. These results are promising for these lipid and chitosan-based vesicles in wound therapy.

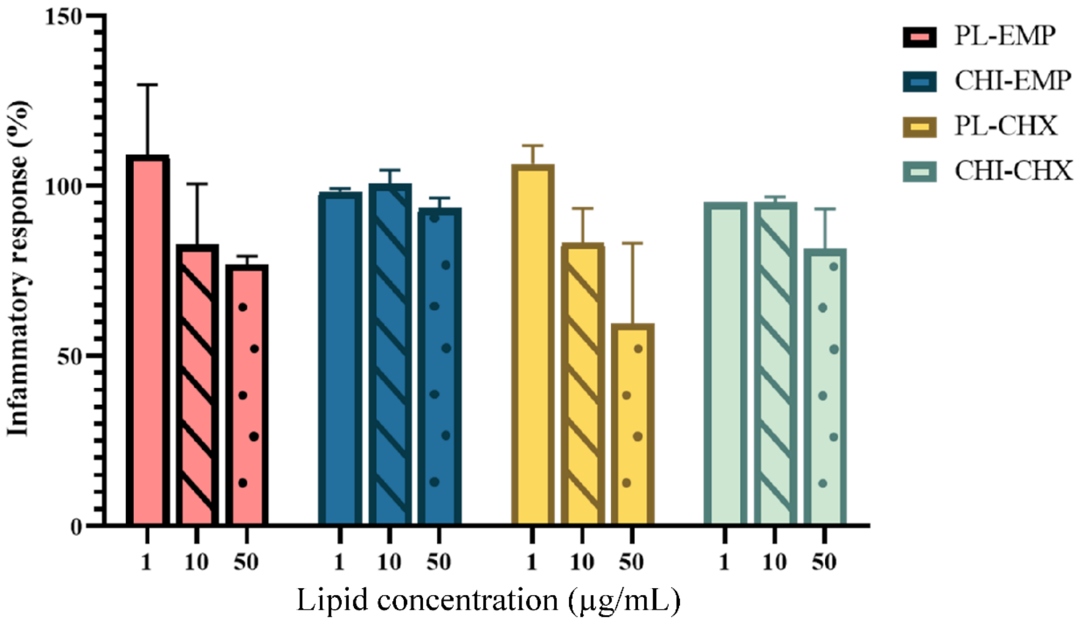

Figure S1. Evaluation of anti-inflammatory activity of vesicles on RAW 264.7 cells. Three different concentrations were tested, namely 1 (no pattern), 10 (stripes) and 50 (dots) µg/mL lipid and the results are presented as inflammatory response of treated cells compared to control (100%). All results are expressed as means with their respective SD (n = 2).

PL-EMP = plain, empty vesicles, CHI-EMP = empty chitosomes, PL-CHX = plain, CHX-vesicles, CHI-CHX = CHX-chitosomes.

S2. Supplementary methods

S2.1. Stability testing of hydrogels

The stability of hydrogels and vesicles-in-hydrogel were evaluated after storage for two and four weeks after preparation. The texture properties and pH were measured as described in section 3.4.2.

S2.2. Anti-inflammatory activity

Anti-inflammatory activity was determined by LPS-induced NO production in murine macrophages RAW 264.7 cells [88]. Cells were cultured in complete RPMI (containing 10 % fetal bovine serum and 1 % penicillin and streptomycin) and seeded in 24 well plates prior to incubation (37 °C /5 % CO_2) for 24 hours. After incubation, the complete medium was replaced with medium containing LPS (1 μg/mL). The cells were then treated with chitosome suspensions of different concentrations (1, 10 and 50 μg/mL lipid concentration) diluted in LPS (1 μg/mL) containing medium. LPS containing medium and complete medium served as positive and negative controls. The cells were then incubated for another 24 hours and the NO production was evaluated on a UV-vis plate reader (Tecan Trading AG, Männedorf, Switzerland) with Griess reagent (2.5 % phosphoric acid with 1 % sulphanilamide and 0.1 % N-(-1-naphthyl)ethylenediamine) at 540 nm.

Supplementary references

38. Hurler, J.; Žakelj, S.; Mravljak, J.; Pajk, S.; Kristl, A.; Schubert, R.; Škalko-Basnet, N., The effect of lipid composition and liposome size on the release properties of liposomes-in-hydrogel. *Int. J. Pharm.* **2013,** 456, 49-57; https://doi.org/10.1016/j.ijpharm.2013.08.033.

44. Hurler, J.; Engesland, A.; Kermany, B. P.; Škalko-Basnet, N., Improved texture analysis for hydrogel characterization: Gel cohesiveness, adhesiveness, and hardness. *J. Appl. Polym. Sci.* **2012,** 125, 180-188; https://doi.org/10.1002/app.35414.

45. Jøraholmen, M. W.; Basnet, P.; Tostrup, M. J.; Moueffaq, S.; Škalko-Basnet, N., Localized Therapy of Vaginal Infections and Inflammation: Liposomes-In-Hydrogel Delivery System for Polyphenols. *Pharmaceutics* **2019,** 11, 53; https://doi.org/10.3390/pharmaceutics11020053.

88. Cauzzo, J.; Nystad, M.; Holsæter, A. M.; Basnet, P.; Škalko-Basnet, N., Following the Fate of Dye-Containing Liposomes In Vitro. *Int. J. Mol. Sci.* **2020,** 21, 4847; https://doi.org/10.3390/ijms21144847.
